# Influence of an Intermediate Option on the Description-Experience Gap and Information Search

**DOI:** 10.3389/fpsyg.2018.00364

**Published:** 2018-03-28

**Authors:** Neha Sharma, Shoubhik Debnath, Varun Dutt

**Affiliations:** ^1^Applied Cognitive Science Laboratory, Indian Institute of Technology Mandi, Kamand, India; ^2^Interaction Lab, University of Southern California, Los Angeles, CA, United States

**Keywords:** description, experience, investment, intermediate option, Natural Mean Heuristic, rare outcomes, common outcomes

## Abstract

Research shows that people tend to overweight small probabilities in description and underweight them in experience, thereby leading to a different pattern of choices between description and experience; a phenomenon known as the Description-Experience (DE) gap. However, little is known on how the addition of an intermediate option and contextual framing influences the DE gap and people’s search strategies. This paper tests the effects of an intermediate option and contextual framing on the DE gap and people’s search strategies, where problems require search for information before a consequential choice. In the first experiment, 120 participants made choice decisions across investment problems that differed in the absence or presence of an intermediate option. Results showed that adding an intermediate option did not reduce the DE gap on the maximizing option across a majority of problems. There were a large majority of choices for the intermediate option. Furthermore, there was an increase in switching between options due to the presence of the intermediate option. In the second experiment, 160 participants made choice decisions in problems like those presented in experiment 1; however, problems lacked the investment framing. Results replicated findings from the first experiment and showed a similar DE gap on the maximizing option in a majority of problems in both the absence and presence of the intermediate option. Again, there were a large majority of choices for the intermediate option. Also, there was an increase in switching between options due to the presence of the intermediate option. Meta-analyses revealed that the absence or presence of the intermediate option created certain differences in the strength of frequency and recency processes. Also, a single natural-mean heuristic model was able to account for the experimental results across both experiments. We discuss implications of our findings to consequential decisions made after information search.

## Introduction

Warren Buffet, in one of his speeches, stated that “risk comes from not knowing what you’re doing” (Berman, 2014). Investment sector has remained a popular business destination with people investing in stocks and bonds. However, such investment options have their own set of risks, which, for the most part depend upon the number of options and probabilistic returns (Houpt and Border, 2014).

Investment decisions, which are mostly consequential, are likely to be made by gathering information in two formats: description and experience. For example, new investors in the stock market may prefer descriptive knowledge of the investment options, their outcomes, and associated probabilities. However, for experienced stock traders, investment decisions maybe guided by one’s prior experience of making such decisions (Nakov and Nuño, 2015).

Furthermore, often people may be presented with multiple investment options, where certain options are asymmetrically dominated by other options in the list.^1^ These asymmetrically dominated options are referred to as intermediate options. Prior research has shown that the presence of an intermediate option changes people’s preferences (Huber et al., 1982). For example, when people are asked whether they want to eat at a five-star restaurant that is far away, or a three-star restaurant that is nearby, the choice may be for either of the two restaurants (Huber et al., 1982). However, when a third (intermediate) option of a four-star restaurant that takes longer to get to compared to the five-star establishment is added, then people start choosing the five-star restaurant regularly (Huber et al., 1982).

Literature in Judgment and Decision-Making has proposed two paradigms to study people’s decisions: decisions from description and decisions from experience. In decisions from description, probability and outcomes in a problem are described in a textual format and people choose an option they prefer after reading different text descriptions (Kahneman and Tversky, 1979; Tversky and Kahneman, 1992; Hertwig et al., 2004). However, in decisions from experience, people first sample options searching for outcomes and, once satisfied with their sampling, decide the option they prefer (Hertwig et al., 2004).^2^ Sampling of options is costless and sampling could be done any number of times and in any order (Hertwig et al., 2004).

Prior research shows that people make different choices while making decisions from experience and when making decisions from description (Hertwig et al., 2004). When people make experience-based decisions, people tend to underweight the probability of rare outcomes more than these outcomes deserve as per their objective probabilities (Hertwig et al., 2004); however, this behavior is reversed in description-based decisions. In description-based decisions, people tend to overweight the probability of rare outcomes more than these outcomes deserve as per their objective probabilities (Kahneman and Tversky, 1979; Tversky and Kahneman, 1992). Due to this overweighting in decisions from description, people exhibit a fourfold pattern of risk preferences (Tversky and Fox, 1995; Hertwig, 2012). According to this fourfold pattern, people generally risk averse when the probability of winning is high but risk seeking when it is low (Tversky and Fox, 1995; Hertwig, 2012). In addition, people are risk averse when the probability of losing is low but risk seeking when it is high (Tversky and Fox, 1995; Hertwig, 2012). Overall, the difference in weighting of rare outcomes in experience and description causes people to make different choices in these formats and this phenomenon has been term as the Description-Experience (DE) gap (Hertwig and Erev, 2009).

The DE gap has been studied under two-option scenarios across several investigations (Gottlieb et al., 2007; Ungemach et al., 2009; Erev et al., 2010; Abdellaoui et al., 2011; Gonzalez and Dutt, 2011; Hilbig and Glöckner, 2011; Lejarraga et al., 2012). Also, several experience-based models have been proposed that account for people’s experience-based decisions by relying on processes concerning frequency and recency of experiencing outcomes and the magnitude of outcomes (Erev et al., 2010; Hertwig and Pleskac, 2010; Gonzalez and Dutt, 2011). For example, one model called the natural-mean heuristic (NMH) has been shown to account for people’s experience-based decisions based upon their sampling of options across many problems (Hertwig and Pleskac, 2010).

Although the study of DE gap and reasons for this gap have been an active area of research (Hertwig and Erev, 2009), less attention has been given on how adding intermediate options in problems influences people’s decision choices and the DE gap. In addition, little is known on how a problem’s framing, i.e., whether it is a decision to eat at a restaurant or simply a decision without any restaurant framing, influences this gap.

Furthermore, one of the potential influencers of DE gap includes different sampling strategies (Hills and Hertwig, 2010). According to investigators, in decision problems, individuals who transit more frequently between options in experience tend to choose options that underweight rare outcome, increasing the DE gap (Hills and Hertwig, 2010). This behavior is different for individuals who transit less frequently between options. However, it is still unclear how the sampling strategy changes in the presence of intermediate options.

In this paper, across two experiments, we investigate the nature of the DE gap in people’s choices and judgments as a function of the presence or absence of an intermediate option and as a function of the decision problem’s framing. In addition, we also study how people’s sampling strategies in experience-based decisions are influenced by the presence of an intermediate option. In summary, we take the existing literature forward in three different ways. First, we propose the study of DE gap among problems with and without an intermediate option. In our studies, problems have a choice-set size of two or three options and, therefore, in such problems, participants have two or three options to choose between. Second, prior research had mostly confined the problem domain to abstract scenarios (Hills et al., 2013; Frey et al., 2015; Noguchi and Hills, 2016). In our study, we extend the influence of intermediate option on DE gap to both problems with and without a contextual framing. Third, prior research had only proposed to investigate the DE gap in terms of people’s choices. In our studies, we not only study the DE gap in terms of a person’s choices for one of the options; rather, we also investigate the DE gap in terms of a person’s judgment about the percentage of investments across different choice options.

In what follows, we first motivate our hypotheses related to addition of intermediate option and problem framing. Next, we detail two experiments where we tested the influence of intermediate options and problem framing on decision choices. We close the paper by highlighting the implications of our findings to decisions made from information search.

### Background

Although little is known on the influence of intermediate options and problem framing on the DE gap; however, researchers have investigated how choice-set sizes influence people’s information search and subsequent decisions (Hills et al., 2013). It has been found that an increase in the number of options in problems influences information search with participants taking more samples overall and fewer samples per option with larger set sizes (Hills et al., 2013).

Furthermore, research has studied information search and decision choices due to variations in choice-set sizes among people from different age groups (He and Wu, 2016). Results suggest that younger and older adults are relatively similar in how they searched for information and make subsequent choices when faced with two-option problems; however, information search and subsequent choice are different between younger and older adults when the choice-set size increases (Frey et al., 2015). Another study has investigated how choice-set size influenced people’s preferences that were indicative of inequity aversion (He and Wu, 2016). These researchers found that inequity aversion was influenced by choice-set size and that that the variance in inequity aversion increased with the range of choice sets.

Also, it has been found that experience-based decisions favor riskier options in larger choice-set size problems compared to smaller set-size problems, diminishing the DE gap (Noguchi and Hills, 2016). It has been demonstrated that risk-taking behavior increases as the choice-set size increases, thereby promoting choices with riskier positive options in experience-based decisions; but, not in the description-based decisions (Noguchi and Hills, 2016).

Although the influence of choice-set size on the DE gap has been studied, research is needed that investigates the influence of intermediate options and problem framing on the DE gap. In this paper, we undertake this research using lab-based experiments. Specifically, across two experiments, we study problems with or without an intermediate option and those that are with or without a contextual framing. We investigate the influence of intermediate options and problem framing on the DE gap as well as people’s search strategies. Furthermore, we also investigate whether experience-based models (like NMH) are able to account for experimental results.

### Hypotheses

In decisions from description, people make choices as if they overweight the probability of rare outcomes; and, in case of decisions from experience, in contrast, people make choices as if they underweight the probability of rare outcomes (Hertwig et al., 2004). As per prior investigations (Dutt and Gonzalez, 2012), possible causes of the underweighting of rare outcomes in experience are recency and frequency of sampled information, magnitude of rare outcomes, and the number of samples of different options (sample size) (Hertwig and Erev, 2009; Hertwig, 2012).

When people make decisions from experience, because of reliance on recency and frequency of information, they are likely to underweight rare outcomes and chose options that maximize experienced expected values (Gonzalez and Dutt, 2011; Lejarraga et al., 2012). This underweighting and maximization processes will likely cause different choices in problems with and without an intermediate option. If a problem consists of a binary choice, where one option is variable with a non-zero rare outcome and the other option is fixed with a constant outcome, then people would likely make a choice for the constant option due to recency and frequency mechanisms that underweight the non-zero rare outcome. Now, if the same problem consists of an additional intermediate option, where the probability of non-zero outcome in the intermediate option is high and its magnitude is higher than that of the fixed option, then people would likely underweight the non-zero rare outcome and discount the variable option. Next, while choosing between the fixed option and intermediate option, people would tend to overweight the high probability of non-zero outcome in the intermediate option and choose the intermediate option as doing so would maximize their rewards. Overall, the addition of the intermediate option is likely to create an asymmetric dominance effect that influences the decision toward the intermediate option.

Furthermore, if a problem consists of a binary choice, where one option is variable with a non-zero frequent outcome and the other option is fixed with a constant outcome, then people would likely make a choice for the variable option due to recency and frequency mechanisms that underweight the zero-valued rare outcome in the variable option. Moreover, if people experience the outcomes in the same way across different problem framing, then one would expect that the underweighting due to frequency and recency effects would be present in similar ways across both problems, with and without a contextual framing. In summary, according to recency and frequency reliance (Dutt and Gonzalez, 2012), the problem framing should not change the way people make final choices in problems. However, if people do not see outcomes in similar ways across different problem framings, then one expects different final choices across different problem framings.

Furthermore, literature has revealed two types of switching strategies among people while they search for information (Hills and Hertwig, 2010): comprehensive and piecewise. In comprehensive strategy, people search for one option before moving to the other option and switch little between options; however, in piecewise, people search for both options one after the other and they switch a lot more between options compared to the comprehensive strategy. One expects the switching strategy to be predominantly piecewise when the intermediate option is present compared to it being a mix of piecewise and comprehensive when the intermediate option is absent. That is because, when there is an intermediate option in addition to the variable and fixed options to choose between, people would likely shift from variable and fixed options to the intermediate option. That is expected because of the underlying frequency and recency processes and maximization of outcomes.

Furthermore, literature has proposed several computational models for accounting for people’s decision from experience (Erev et al., 2010; Hertwig and Pleskac, 2010; Gonzalez and Dutt, 2011, 2012). One of the popular models that has been found to account for people’s experiential decisions is Natural Mean Heuristic (NMH) (Hertwig and Pleskac, 2010). The NMH model computes the natural mean of sampled outcomes and chooses the option with the higher natural mean. Thus, the NMH model incorporates the sample size, outcome magnitude, and outcome frequency as part of its decision process. As these processes have been found to influence people’s experiential decisions (Dutt and Gonzalez, 2012), we expect the NMH model to capture people’s final choices both in the presence or absence of the intermediate option in problems with and without a contextual framing. We test these expectations across two experiments in the upcoming sections.

## Experiment 1: the DE Gap in Problems With an Intermediate Option and Investment Framing

In this experiment, we test the influence of intermediate option on DE gap in rare and common problems involving an investment framing.

### Method

#### Experimental Design

**FIGURE 1:**
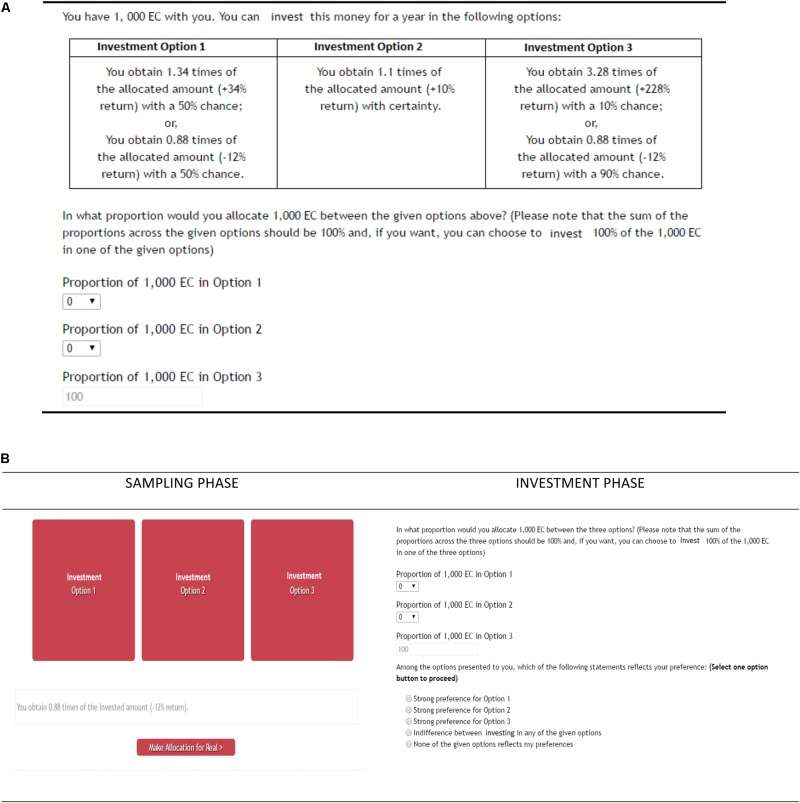
The investment task used in the description and experience conditions. **(A)** The description condition, where the probabilities and outcomes in different options were textually described. Participants were asked to read these text descriptions and decide the percentage of investment in different options. Participants were also asked to provide their choice for one of the options for real. **(B)** The experience condition, where the probabilities and outcomes in different options were experienced. First, participants were asked to sample options presented on a computer screen (sampling phase). Sampling options did not affect participant payoffs and participants were free to sample options as many times and in any order they desired. Once participants were satisfied with their sampling, they were asked to provide their choice for one of the options for real (investment phase).

The experiment involved two between-subject conditions: experience (*N* = 60) and description (*N* = 60). These conditions differed in the format of presentation of options, descriptive or experiential. In description, as shown in **Figure 1A**, investment options were presented to participants as a text description. Based upon this description, participants were asked to allocate their endowment to different options. Participants were also asked to indicate the option they preferred. In experience, as shown in **Figure 1B** (left side), participants were first asked to sample investment options (presented as blank buttons; sampling phase). During the sampling phase, every time an investment option was selected by pressing the corresponding button, participants could see an outcome generated based upon the associated probability. Sampling of investment options was costless and participants were free to sample options in any order and as many times as they desired. At any time during the sampling phase, participants could click the “Make Investments” button. Clicking the “Make Investments” button terminated the sampling phase and moved participants to the investment phase as shown in **Figure 1B** (right side). In the investment phase, participants were asked to make a final allocation to different investment options and indicate their preferred option.

Within the description and experience conditions, participants were randomly assigned to one of two between-subjects problems: two-option problems (*N* = 30) and three-option problems (*N* = 30). The two-option problems contained only two investment options to search or choose between (choice-set size = 2): an option with a fixed return on investment (1.1 return on the invested amount with certainty; expected value = 1.1); and, an option with a variable (probabilistic) return on investment. In one of the two-option problems (common-event, CE), the variable option had a high probability (0.8) value associated with a high (H) outcome (1.18 return on the invested amount); whereas, in the other two-option problem (rare-event, RE), the variable option had a low probability (0.1) associated with the H outcome (3.28 return on the invested amount). Across both the two-option problems (CE and RE), in the variable option, the low (L) outcome (0.88) always occurred with a probability that was 1.0 minus the probability of the H outcome. The expected value of the variable option in the CE and RE two-option problems was 1.12.

Similarly, in the three-option problems, there were three investment options (including an intermediate option) to search and choose between (choice-set size = 3). In each of the three-option problem, participants were presented with three investment options: fixed option, variable option, and intermediate option. The definition of the fixed and variable options was the same as that in the two-option problems (above). The intermediate investment option was defined in the following way: Obtain 1.34 times the invested amount with a 50% chance and get 0.88 times the invested amount with a 50% chance (expected value = 1.11). The expected value of the intermediate option was less than that of the variable option. However, the expected value of the intermediate option was greater than that of the fixed option. Thus, the intermediate option was in between the variable and fixed options and termed the intermediate option. Also, based on the expected values, the variable option was maximizing in all three-option problems. Overall, the nature of outcomes and probabilities in different CE and RE problems were like those described in the literature (Huber et al., 1982). For our statistical analyses, we assumed an alpha level of 0.05 and a power level of 0.80.

#### Participants

This study was carried out in accordance with the recommendations of ethics committee at Indian Institute of Technology Mandi with a written informed consent from all participants. Participation was voluntary and all participants gave consent before starting their study. One hundred and twenty students at Indian Institute of Technology Mandi, India, participated in the study. Ages ranged from 18 to 45 years (mean = 25 years; *SD* = 7 years). Sixty-two percent of participants were males and rest females. Ninety-eight percent of participants had undergraduate degrees and the rest were pursuing Ph.D. degrees. Most participants (85%) had never traded on the stock market. Participants were given a maximum of 30 min for finishing the study and all participants completed their study within this time. Participants were paid a flat participation fee of USD 0.8 for their participation. Participants were told that the top-10 performing participants across problems will enter a lucky draw and one participant will be randomly picked and paid USD 7.5. At the end of the study, a lucky draw was held, and one participant was paid USD 7.5.

#### Procedure

Participants were randomly assigned to different conditions and they were told that they were investors and that they could invest their money in different investment options for a period of 1 year. The order of presentation of the CE and RE problems and the order of presentation of options within each of these problems were randomized for each participant across different two-option and three-option problems in description and experience conditions. Across both description and experience conditions, participants first read instructions that appeared on a computer terminal. The experimenter answered any question before the participant could begin the experiment. The investment task used a fictitious currency called “EC,” where 1 INR = 10 EC. For each problem, participants were asked to assume they were endowed with 1,000 EC, which they had to invest in different options in a problem. The participants, however, were not shown outcomes obtained in a problem upon making investments (this information was revealed to participants only at the end of their study, i.e., when they had played the two problems). Based upon investments made, a participant could end-up gaining or losing money. Participants’ goal in the study was to invest money across different options such that their return on investment was maximized.

### Results

#### The DE Gap in Two- and Three-Option Problems

We performed two-way between-subjects ANOVAs to compare the effect of conditions (description, experience) and options (two, three) on the allocation and choice preferences for the variable (maximizing) and intermediate options. There were separate ANOVAs performed for the CE and RE problems.

**FIGURE 2:**
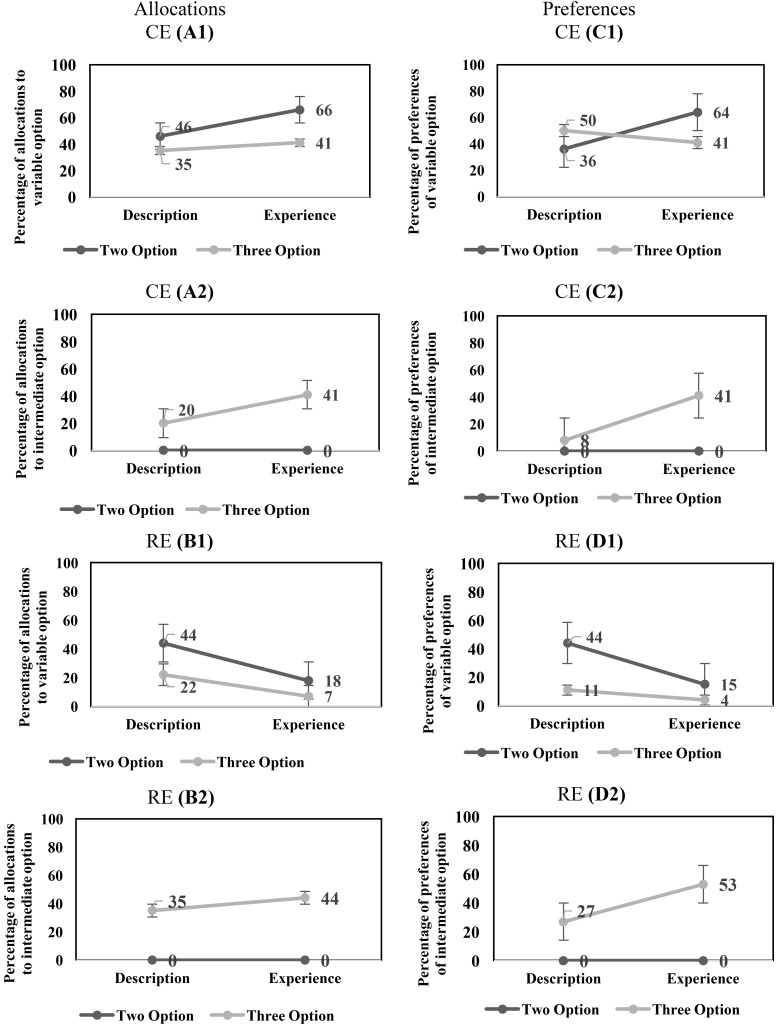
**(A1,A2)** The influence of conditions and options on the percentage of allocation to the variable option **(A1)** and intermediate option **(A2)** in CE problems. **(B1,B2)** The influence of conditions and options on the percentage of allocation to the variable option **(B1)** and intermediate option **(B2)** in RE problems. **(C1,C2)** The influence of conditions and options on the percentage of preferences of the variable option **(C1)** and intermediate option **(C2)** in CE problems. **(D1,D2)** The influence of conditions and options on the percentage of preferences of the variable option **(D1)** and intermediate option **(D2)** in RE problems.

For the CE problems, there was a significant main-effect of conditions on the percentage of allocation to the variable option [description: 41% < experience: 54%; *F*(1,116) = 4.19, *p* = 0.04, $\eta_{p}^{2}$ = 0.035]. In addition, there was a significant main-effect of options on the percentage of allocations to the variable option [two-option: 56% > three-option: 38%; *F*(1,116) = 7.887, *p* = 0.01, $\eta_{p}^{2}$ = 0.064]. However, the interaction effect of conditions and options on the percentage of allocations to the variable option was not significant [*F*(1,116) = 1.081, *p* = 0.30, $\eta_{p}^{2}$ = 0.009]. **Figure 2(A1)** shows the influence of conditions and options on the percentage of allocation to the variable in the CE problems.

Next, we investigated the effects of conditions and options on the intermediate option in the CE problems. In the CE problems, there was a significant main-effect of conditions on the percentage of allocation to the intermediate option [description: 10% < experience: 21%; *F*(1,116) = 5.82, *p* = 0.02, $\eta_{p}^{2}$ = 0.05]. There was a significant main-effect of options on the percentage of allocations to the intermediate option [two-option: 0% < three-option: 31%; *F*(1,116) = 49.20, *p* < 0.001, $\eta_{p}^{2}$ = 0.30]. Furthermore, there was a significant interaction effect of conditions and options on the percentage of allocations to the intermediate option [*F*(1,116) = 5.82, *p* = 0.02, $\eta_{p}^{2}$ = 0.05].

**Figure 2(A2)** shows the influence of conditions and options on the percentage of allocation to the intermediate option in the CE problems.

In the RE problems, there was a significant main-effect of conditions on the percentage of allocations to the variable option [description: 33% > experience: 13%; *F*(1,116) = 20.06, *p* < 0.001, $\eta_{p}^{2}$ = 0.15]. In addition, there was a main-effect of options on the percentage of allocations to the variable option [two-option: 31% > three-option: 15%; *F*(1,116) = 13.20, *p* < 0.001, $\eta_{p}^{2}$ = 0.10]. However, the interaction effect of conditions and options on the percentage of allocations to the variable option was not significant [*F*(1, 116) = 1.65, *p* = 0.20, $\eta_{p}^{2}$ = 0.01]. **Figure 2(B1)** shows the influence of conditions and options on the percentage of allocation to the variable option in the RE problems.

Next, we investigated the effects of conditions and options on the intermediate option in the RE problems. In the RE problems, the main-effect of conditions on the percentage of allocation to the intermediate option was not significant (description: 18% ∼ experience: 22%; [*F*(1,116) = 1.24, *p* = 0.27, $\eta_{p}^{2}$ = 0.01]. There was a significant main-effect of options on the percentage of allocations to the intermediate option [two-option: 0% < three-option: 40%; *F*(1,116) = 94.49, *p* < 0.001, $\eta_{p}^{2}$ = 0.45]. Furthermore, the interaction effect of conditions and options on the percentage of allocations to the intermediate option was not significant [*F*(1,116) = 1.24, *p* = 0.26, $\eta_{p}^{2}$ = 0.01]. **Figure 2(B2)** shows the influence of conditions and options on the percentage of allocation to the intermediate option in the RE problems.

In the CE problems, the DE gap on the variable option was negative and similar between the two-option problems and three-option problems. Furthermore, the DE gap on the intermediate option was not present in the two-option problems; however, like the variable option, it was negative in the three-option problems. In the RE problems, the DE gap on the variable option was positive and similar across the two-option and three-option problems. Furthermore, the DE gap on the intermediate option was not present in both two-option and three-option RE problems. Across both experience and description conditions in CE and RE problems, the percentage of allocations to the variable option decreased and to the intermediate option increased from the two-option problems to the three-option problems. Overall, these results show that the DE gap on the allocation percentages to the variable option in the two-option and three-option CE and RE problems was similar to that reported in literature (Hertwig et al., 2004).

As conventionally done in literature (Hertwig et al., 2004), next, we evaluated the DE gap in terms of choice preferences for the variable option. For the CE problems, the main-effects of conditions and options on the percentage of preferences of the variable option were not significant [conditions: description: 43% ∼ experience: 53%; *F*(1,104) = 1.05, *p* = 0.31, $\eta_{p}^{2}$ = 0.01; options: two-option: 50% ∼ three-option: 46%; *F*(1,104) = 0.22, *p* = 0.64, $\eta_{p}^{2}$ = 0.002]. However, there was a significant interaction effect of conditions and options on the percentage of preferences of the variable option [*F*(1,104) = 3.70, *p* = 0.05, $\eta_{p}^{2}$ = 0.034]. **Figure 2(C1)** shows the influence of conditions and options on the percentage of preferences to the variable option in the CE problems.

Next, we investigated the effects of conditions and options on the percentage of preferences of intermediate option in the CE problems. There was a significant main-effect of conditions on the percentage of preferences of the intermediate option [description: 04% < experience: 21%; *F*(1,116) = 10.66, *p* < 0.001, $\eta_{p}^{2}$ = 0.08]. There was a significant main-effect of options on the percentage of preferences of the intermediate option [two-option: 0% < three-option: 25%; *F*(1,116) = 20.90, *p* < 0.001, $\eta_{p}^{2}$ = 0.15]. Furthermore, there was a significant interaction effect of conditions and options on the percentage of preferences of the intermediate option [*F*(1,116) = 10.66, *p* < 0.001, $\eta_{p}^{2}$ = 0.08]. **Figure 2(C2)** shows the influence of conditions and options on the percentage of preferences to the intermediate option in the CE problems.

In the RE problems, there was a significant main-effect of conditions on the percentage of preferences of the variable option [description: 28% > experience: 09%; *F*(1,106) = 7.18, *p* = 0.01, $\eta_{p}^{2}$ = 0.06]. In addition, there was a main-effect of options on the percentage of preferences of the variable option [two-option: 30% > three-option: 07%; *F*(1,106) = 10.74, *p* < 0.001, $\eta_{p}^{2}$ = 0.09]. However, the interaction effect of conditions and options on the percentage of preferences of the variable option was not significant [*F*(1,106) = 2.69, *p* = 0.10, $\eta_{p}^{2}$ = 0.03]. **Figure 2(D1)** shows the influence of conditions and options on the percentage of preferences of the variable option in the RE problems.

Next, we investigated the effects of conditions and options on the intermediate option in the RE problems. There was a significant main-effect of conditions on the percentage of preferences of the intermediate option [description: 14% < experience: 27%; *F*(1,116) = 4.64, *p* = 0.03, $\eta_{p}^{2}$ = 0.04]. There was a significant main-effect of options on the percentage of preferences of the intermediate option [two-option: 0% < three-option: 40%; *F*(1,116) = 41.76, *p* < 0.001, $\eta_{p}^{2}$ = 0.27]. Furthermore, there was a significant interaction effect of conditions and options on the percentage of preferences of the intermediate option [*F*(1,116) = 4.64, *p* = 0.03, $\eta_{p}^{2}$ = 0.04]. **Figure 2(D2)** shows the influence of conditions and options on the percentage of preferences of the intermediate option in the RE problems.

In the CE problems, the DE gap on the variable option reversed in sign between the two-option problems (negative) and three-option problems (positive). Furthermore, the DE gap on the intermediate option was not present in the two-option problems; however, it was negative in the three-option problems. In the RE problems, the DE gap on the variable option was positive and similar across both the two-option and three-option problems. Furthermore, the DE gap on the intermediate option was not present in the two-option RE problems; however, unlike the variable option, it was negative in the three-option RE problems. Across description and experience conditions, the percentage of preferences of the variable option either remained constant (CE problem) or decreased (RE problem) from the two-option problems to the three-option problems; however, the percentage of preferences of the intermediate option increased from the two-option problems to the three-option problems across both CE and RE problems. Overall, these results show that the DE gap on preferences of the variable option was similar to that reported in literature for two-option CE and RE problems (Hertwig et al., 2004). However, the nature of the DE gap reversed in the three-option CE problem (it continued to agree with literature for the three-option RE problem).

#### Influence of Choice-Set Size on Switching Behavior

The way people switch between options is likely to be influenced by choice-set size. As our next step, we analyzed participant’s switching behavior between the options presented to her during sampling. A switch ratio was calculated for each participant by assuming the ratio between the participant’s number of switches between options in a problem and the maximum number of allowable switches (i.e., *n* - 1, where *n* is the total number of samples in a problem). We also estimated the median number of switches per problem.

**FIGURE 3:**
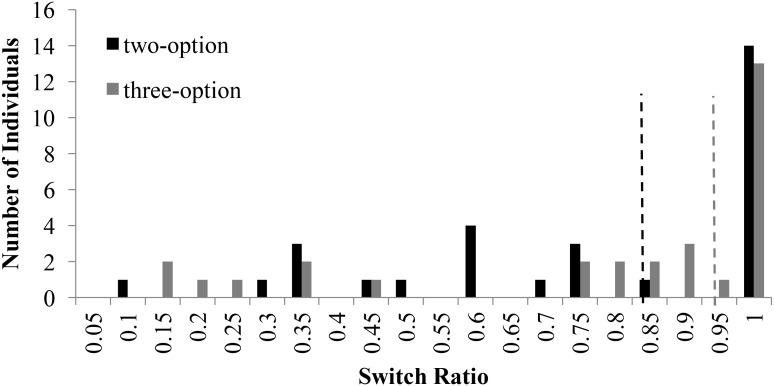
Switch ratio versus number of individuals across options in the CE problem. The dashed line in black represents the median switch ratio (=0.83) for the two-option CE problem; whereas, the dashed line in grey represents the median switch ratio (=0.93) for the three-option CE problem.

**Figure 3** shows the distribution of switch-ratios in two- and three-option CE problems. For the two-option CE problems, the plot was bimodal and showed two peaks at 0.60 and 1.00 switch ratios. This bimodal nature was in accordance with results in literature (Hills and Hertwig, 2010), where people with 1.0 switch ratio followed the piecewise strategy and those with a 0.60 switch ratio followed more like a comprehensive strategy. However, for the three-option CE problem, the peak at 0.60 shifted to 0.90, moving comprehensive strategists to piecewise strategists. In fact, there was a forward shift in the median between two-option problems and three-option problems. Overall, the three-option problems caused participants to switch more between options compared to the two-option problems.

**FIGURE 4:**
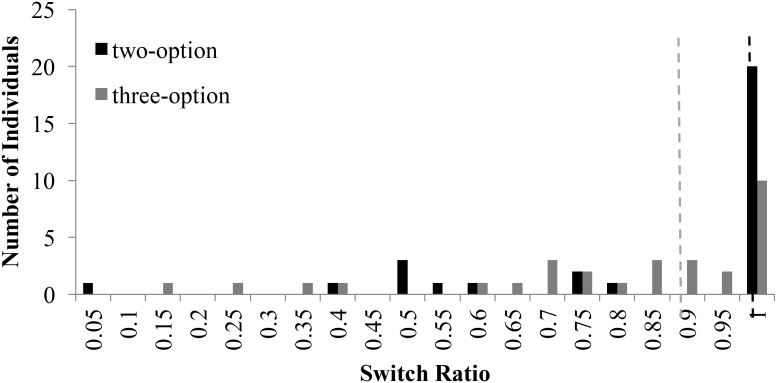
Switch ratio versus number of individuals across options in the RE problems. The dashed line in black represents the median value (=1.0) for two-option problems; whereas, the dashed line in grey represents the median value (=0.85) for three-option problems.

**Figure 4** shows the distribution of switch-ratios in two- and three-option RE problems. For the two-option RE problem, the switch ratio histogram again showed two peaks at 0.50 and 1.00, exhibiting a bi-modal distribution. This bi-modal nature is in accordance to prior literature (Hills and Hertwig, 2010), where people with 1.0 switch ratio followed the piecewise strategy and those with a 0.60 switch ratio followed more like a comprehensive strategy. However, for the three-option RE problem, a prominent peak was observed at 1.00 switch ratio with a general shift in the second mode to higher values between 0.85 and 0.95. The shift in mode from 0.5 to values between 0.85 and 0.95 indicated the movement of comprehensive strategist to piecewise strategists.

For both the common- and rare-event problems, the histograms plots reveal several conclusions. First, the right shift of intermediate mode could be attributed to the fact that, as number of options increased, people likely relied on recency of encountered outcomes in options causing them to forget outcomes that were distant and less frequent. Therefore, participants tended to sample the options more in a piecewise manner rather than in a comprehensive manner when the number of options increased from two to three. Additionally, for the RE problems, the modality of the histogram plot changed from a bi-modal plot to a multi-modal plot as the number of options increased from two to three. In fact, in RE problems, the intermediate mode seemed to have shifted right and split-up into several peaks between switch-ratios of 0.85 and 0.95. This shifted pattern in RE problems was like the one shown by participants in the CE problems.

#### Limited Information Search

Experience-based decisions are influenced by the number of times options containing rare outcomes are sampled. For example, in the RE problem, if the maximizing option has an outcome 3.28 that occurs with a 10% chance and an outcome 0.88 that occurs with a 90% chance, then one expects to see the outcome 3.28 once on average in every 10 samples from this option. However, if a participant takes less than 10 samples of this option, then this participant may not encounter the outcome 3.28 at all and she will likely consider this option to only provide a non-maximizing 0.88 return and avoid investing in this option. Thus, due to small samples, participants are likely to underweight rare outcomes.

**FIGURE 5:**
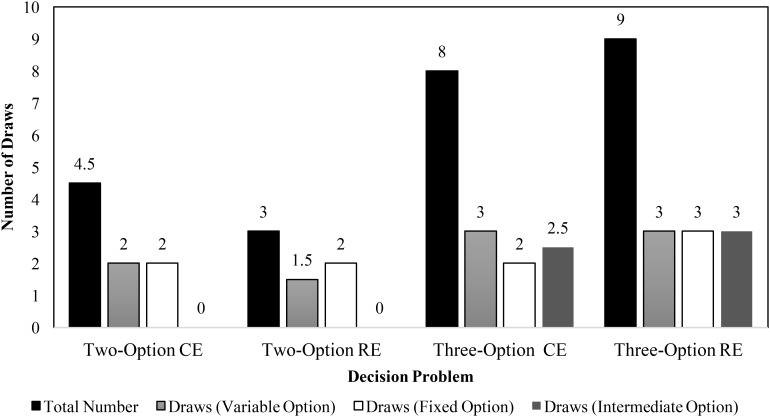
Median number of draws during sampling in two- and three-option CE and RE problems in the experience condition.

To investigate underweighting of rare outcomes due to small samples, we analyzed the median sample size across different problems. **Figure 5** displays the median number of draws of different options in CE and RE problems in the experience condition. In the two-option problems, the median values were similar to those reported in literature (Hertwig et al., 2004; Weber et al., 2004). Furthermore, as seen in **Figure 5**, the median number of draws across three-option problems were similar to or higher than those across the two-option problems. The total number of sample draws were greater in the three-option problems compared to the two-option problems. Furthermore, participants performed a number of draws of the intermediate option in three-option problems. These observations agree with those reported in literature (Hills et al., 2013) and they likely cause participants to increase their percentage allocations or preferences for the intermediate option.

#### Frequency Effects

Next, in each problem, we analyzed the choices for the rare and high outcomes in the variable and intermediate options, respectively, for two groups: Those who saw the rare or high outcomes less frequently than expected and those who saw the rare or high outcomes as or more frequently than expected (**Table 1**).

**Table 1:** Percentage of respondents in the experience condition who selected the option involving the rare or high outcomes in investment problems as a function of how often these rare or high outcomes were encountered during sampling.

Decision problem	Options				Percentage choosing variable/intermediate option	
	Variable	Fixed	Intermediate	Rare/high outcome	Encountered as frequently as or more frequently than expected	Encountered less frequently than expected
Two-option CE	1.18, 0.8; 0.88, 0.2	1.1, 1		0.88, 0.2 (Rare)	30 (03/10)	80 (16/20)
Three-option CE	1.18, 0.8; 0.88, 0.2	1.1, 1		0.88, 0.2 (Rare)	43 (03/07)	39 (09/23)
			1.34, 0.5; 0.88, 0.5	1.34, 0.5 (High)	63 (10/16)	14 (02/14)
Two-option RE	3.28, 0.1; 0.88, 0.9	1.1, 1		3.28, 0.1 (Rare)	17 (01/06)	13 (03/24)
Three-option RE	3.28, 0.1; 0.88, 0.9	1.1, 1		3.28, 0.1 (Rare)	25 (01/04)	00 (00/26)
			1.34, 0.5; 0.88, 0.5	1.34, 0.5 (High)	81 (13/16)	21 (03/14)

In the two-option CE problem, when the rare outcome (0.88) was encountered less frequently than expected, 80% of participants selected the variable option. However, when the rare outcome (0.88) was encountered as or more frequently than expected, only 30% of participants selected the variable option. Interestingly, this pattern reversed across the two frequency groups in the three-option CE problem: About 39 and 43% of participants selected the variable option when the rare outcome (0.88) was encountered less frequently than expected and as or more frequently than expected, respectively. This reversal in patterns between the two-option and three-option CE problems likely accounts for the reversal of the DE gap across these problems. As per our expectation, in the three-option CE problem, when the high outcome (1.34) was encountered as or more frequently than expected, about 63% of participants selected the intermediate option. In contrast, only 14% of participants selected the intermediate option when the high outcome in this option was encountered less frequently than expected. These observations account for the increase in the intermediate option choices in three-option CE problems.

In the two-option RE problem, when the rare outcome (3.28) was encountered less frequently than expected, only 13% of participants selected the variable option. However, in agreement with frequency reliance, when the rare outcome (3.28) was encountered as or more frequently than expected, about 17% of participants selected the variable option. This pattern of choices was intensified in the three-option RE problem: 0% of participants selected the variable option with the rare outcome (3.28) when this outcome was encountered less frequently than expected; however, 25% of participants selected the variable option when the rare outcome (3.28) was encountered as or more frequently than expected. Furthermore, 81% of participants selected the intermediate option when the high outcome (1.34) was encountered as or more frequently than expected; however, only 21% of participants selected the intermediate option when the high outcome (1.34) was encountered less frequently than expected. The similarity of patterns between two-option and three-option RE problems likely account for the similarity in the DE gap across these problems. Also, the effect of frequency on intermediate option likely accounts for the increase in the intermediate option choices in three-option RE problems.

#### Recency Effects

While making experience-based decisions, participants need to update their impression of an option by combining newly obtained outcomes with the outcomes of the previous draws (Hertwig et al., 2004). This behavior can give rise to recency effects even in large samples that involve judgments in which recently sampled outcomes receive greater weight than prior sampled ones (Hertwig et al., 2004).

To verify the presence of recency effects, we split the draws during sampling phase into two halves. Then, across each half, we calculated the average payoff for each option. By calculating the average payoff for the first and second halves in each option, we also accounted for the occurrence of the rare outcomes in both halves since the average payoff accounts for both the magnitude and the frequency of outcomes on different options. Next, we compared each participant’s prediction of final choice in a problem based on average payoffs and calculated how many of these predictions coincided with participant’s actual final choices. As shown in **Table 2**, recency was absent in the two-option CE and RE problems; however, the recency effect was 23% (60–37%) and 33% (83–50%), respectively, in the three-option CE and RE problems. Thus, most likely the presence of recency and the high outcome (1.34) on intermediate option in three-option problems caused an increase in the proportion of choices for the intermediate option in these problems compared to the two-option problems.

**Table 2:** Recency effects in two- and three-option common and rare investment problems in the experience condition.

Decision problem	Percentage of final choices that coincided with predicted choice	
	First half	Second half
Two-option CE	70 (21/30)	70 (21/30)
Three-option CE	37 (11/30)	60 (18/30)
Two-option RE	70 (21/30)	70 (21/30)
Three-option RE	50 (15/30)	83 (25/30)

### Natural Mean Heuristic (NMH) Model

The sample size, frequency of experiencing outcomes, and magnitude of experienced outcomes likely influences the DE gap (Hertwig and Erev, 2009; Gonzalez and Dutt, 2011, 2012; Dutt and Gonzalez, 2012). The NMH model accounts for all these effects by computing the natural mean of sampled outcomes on different options and choosing the option with a higher sample mean (Hertwig and Pleskac, 2010). Specifically, the NMH model (Hertwig and Pleskac, 2010) involves the following steps: (1) Calculating the natural mean of observed outcomes for each option by summing, separately for each option, all *n* experienced outcomes and then dividing by *n*; (2) Choosing the option with the highest natural mean. If sample size, outcome magnitude, and outcome frequency influence people’s choices, then NMH model should be able to account for these choice decisions.

**FIGURE 6:**
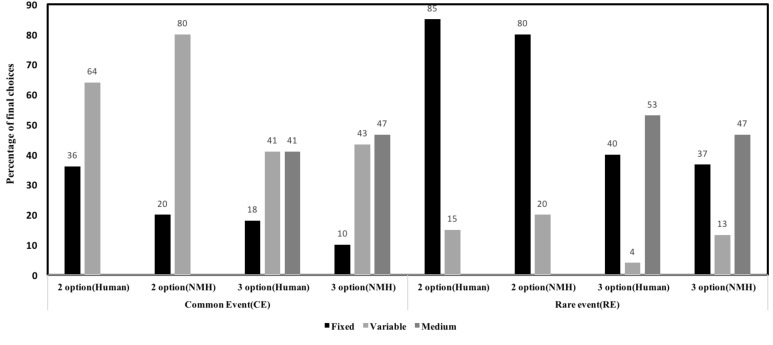
Percentage of final choices for each option by human participants and NMH model in two- and three-option CE and RE problems.

**Figure 6** shows model comparison between NMH model and human data. As shown in the **Figure 6**, the NMH model seems to accurately capture choice preferences in human data. For the two-option CE problem, the mean-squared deviation (MSD) between human data and model across the fixed and variable options was 0.058. For the three-option CE problem, the MSD between human data and model across different options was 0.0098. For the two-option RE problem, the MSD between human data and model across the different options was 0.0056. In three-option RE problem, the MSD between human data and model data across the different options was 0.0144. Overall, the model was able to account for effects in human data due to the influence of conditions and options.

### Discussion

In this experiment, we investigated the influence of choice-set size using problems with rare and common outcomes in an investment framing. More specifically, we evaluated the influence of options (two or three) and conditions (experience or description) on the percentage of allocations and preferences across both the rare and common event problems in an investment framing.

Overall, the DE gap on preferences of the variable option was similar to that reported in the literature for two-option CE and RE problems and three-option RE problem (Hertwig et al., 2004). The existence of the DE gap in the three-option RE problem does not agree with results reported in Noguchi and Hills (2016), where this gap had disappeared. However, the nature of the DE gap reversed in the three-option CE problem compared to that reported in literature (Hertwig et al., 2004). Furthermore, there was an increase in the percentages of preferences of the intermediate option in three-option problems compared to two-option problems. The percentage of preferences of the variable option either remained constant (CE problem) or decreased (RE problem) from the two-option problems to the three-option problems.

One likely reason for the reversal of DE gap in the three-option CE problem could be on account of the reversal in the frequency effects. As explained above, in the three-option CE problem, about 39 and 43% of participants selected the variable option when the rare outcome (0.88) was encountered less frequently than expected and as or more frequently than expected, respectively. These proportions were different from those observed in the two-option CE problem, where there was no reversal in the DE gap. It seems that people did not get influenced by the high outcome (1.18) in the variable option when it was observed more frequently than expected because there was a higher outcome (1.34) on the intermediate option that drove their choices. This reasoning also likely led to the proportion of variable option choices in three-option CE problem to be constant from the two-option problem to the three-option problem.

Second, we found that there was an increase in the percentage of preferences for the intermediate option across all problems. One likely reason for this finding could be the magnitude of the high outcome (1.34) on the intermediate option in three-option problems, large number of samples of the intermediate option in three-option problems, and excessive reliance on recency in three-option problems compared to two-option problems. In the RE problem, although the magnitude of the high outcome on the variable option (3.28) was greater than that on the intermediate option (1.34); the 3.28 outcome occurred rarely and much less frequently than the 1.34 outcome. Thus, on account of recency and larger number of samples of the intermediate option, a large percentage of preferences for the intermediate option. These effects would also be true for the CE problem, where the magnitude of the high outcome on intermediate option (1.34) was much greater than that of the high outcome on the variable option (1.18).

Our results also showed that the choice-set size influenced the distribution of switching behavior. The peak switch ratio for comprehensive switchers moved toward that of piecewise switchers as the number of options increased from two to three. The change in switching behavior could also be attributed to recency and frequency effects: Due to the reliance on either recency or frequency of high outcomes on options, people tended to compare options more rather than restricting themselves to one of the options for a long time.

The NMH model (Hertwig and Pleskac, 2010), incorporating the combined effects of sample size, outcome frequency, and outcome magnitude, was able to account for the findings in this experiment across all problems. As the NMH model could accurately account for human observations, one can conclude that the sample size, frequency, and magnitude of outcomes played a role in explaining effects on preferences due to options and conditions.

In this experiment, we found that conditions and options influenced people’s judgment and choices for different options. However, problems used in this experiment possessed an investment framing. One could argue that the investment framing may also be responsible for our results. Thus, once the investment framing is removed, these effects would also disappear. To test the role of the problem framing on our results, we performed another experiment where we presented participants with problems that were without the investment framing.

## Experiment 2: the DE Gap in Problems With an Intermediate Option and Without Contextual Framing

In Experiment 1, we investigated the influence of an intermediate option in problems possessing an investment framing. However, prior literature (Hertwig et al., 2004) has used abstract problem scenarios to understand choice behavior due to variations in outcome-valence and probabilities. To test whether the effect of an intermediate option on the DE gap was independent of problem framing, we conducted a second experiment involving abstract problems without the investment framing. In this experiment, we investigate the influence of intermediate option across different probability problems as done in the experiment 1; however, problems presented to participants in the current experiment do not possess any contextual framing. Based upon the results of Experiment 1, recency and frequency of information should likely influence people’s decisions irrespective of the problem framing. Thus, we expect a reduction in DE gap on the maximizing option and a shift in sampling strategies in problems with an intermediate option compared to problems without an intermediate option.

### Method

We conducted a laboratory experiment to investigate people’s decision-making from description and experience in problems involving an intermediate option or not and different probabilities of outcomes; however, problems in this experiment lacked the investment framing.

#### Experimental Design

The experiment involved two between-subject conditions: experience (*N* = 80) and description (*N* = 80). In the description condition, options were presented to participants as a text description; however, unlike Experiment 1, these options were presently abstractly as “option 1,” “option 2,” and “option 3” (option 3 was only provided in three-option problems). Based upon this description, participants were asked to allocate 1,000 EC to different options. The word “invest” was changed to “allocate,” which communicated a non-investment framing. Participants were also asked to indicate the option they preferred once they had made their allocations. In experience, participants were first asked to sample options (presented as buttons with labels “option 1,” “option 2,” and “option 3”; sampling phase). During the sampling phase, every time an option was chosen, participants could see an outcome based upon the associated probability in the option. At any time during the sampling phase, participants could click the “Make Allocations” button. Clicking the “Make Allocations” button terminated the sampling phase and moved participants to the allocation phase. In the allocation phase, participants were asked to make a final allocation to different options and indicate which option they preferred. Within each of the description and experience conditions, participants were randomly assigned to one of two between-subjects problems: two-option problems (*N* = 40) and three-option problems (*N* = 40). The two-option problems contained only two investment options to search or choose between (choice-set size = 2); whereas, in the three-option problems, there were three investment options (including an intermediate option) to search and choose between (choice-set size = 3). Within each problem condition, participants were given two problems in a random order. One of these problems was the CE problem, the same one as used in Experiment 1. The other problem was the RE problem, the same one as used in Experiment 1. Other procedures for this experiment remained identical to Experiment 1.

#### Participants

The ethics committee at Indian Institute of Technology Mandi approved the study. This study was carried out in accordance with the recommendations of the ethics committee with a written informed consent from all participants. Participation was voluntary and all participants gave consent before starting their study. One hundred and sixty students at Indian Institute of Technology Mandi, India, participated in the study. Ages ranged from 18 to 45 years (mean = 25 years; *SD* = 7). Seventy-five percent of participants were males and rest females. Ninety-five percent of participants had undergraduate degrees and the rest were pursuing Ph.D. degrees. Participants were given a maximum of 30 min for finishing the study and all participants completed their study within this time. Participants were paid a flat participation fee of USD 0.8 for their participation. Participants were told that, based upon allocations, top-10 performing participants across problems will enter a lucky draw and one participant will be randomly picked and paid USD 7.5.

#### Procedure

Participants were randomly assigned to different conditions and were told that they had to allocate the indicated amount in different options for a unit period. The order of presentation of the CE and RE problems and the order of presentation of options within each of these problems were randomized for each participant across different two-option and three-option problems in description and experience conditions. Across both description and experience conditions, participants first read instructions that appeared on a computer terminal. The experimenter answered any question before the participant could begin the experiment. The allocation task used a fictitious currency called “EC,” where 1 INR = 10 EC. For each problem, participants were asked to assume they were endowed with 1,000 EC, which they had to allocate in different options in a problem. The participants, however, were not shown outcomes obtained in a problem upon making allocation (this information was revealed to participants only at the end of their study, i.e., when they had played the two problems). Based upon allocations made, participants could end-up gaining or losing money. Participants’ goal in the study was to invest money across different options such that their return on allocation was maximized.

### Results

#### The DE Gap in Two- and Three-Option Problems

Like in the first experiment, we performed two-way between-subjects ANOVAs to compare the effect of conditions (description, experience) and options (two, three) on the allocation and choice preferences for the variable (maximizing) and intermediate options. There were separate ANOVAs performed for the CE and RE problems.

**FIGURE 7:**
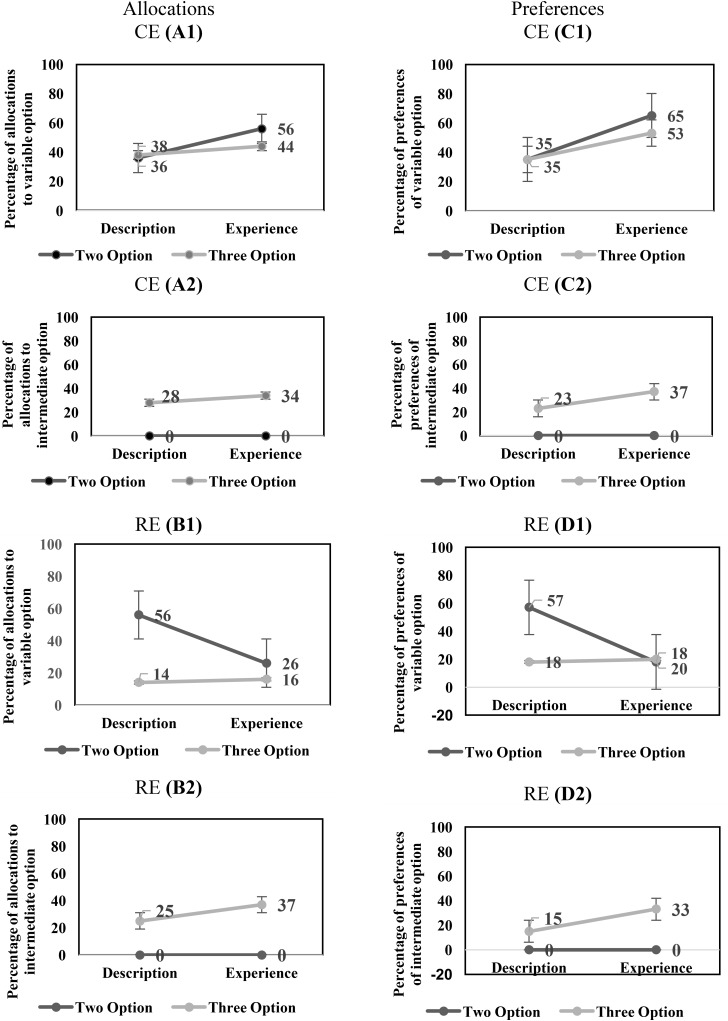
**(A1,A2)** The influence of conditions and options on the percentage of allocation to the variable option **(A1)** and intermediate option **(A2)** in CE problems. **(B1,B2)** The influence of conditions and options on the percentage of allocation to the variable option **(B1)** and intermediate option **(B2)** in RE problems. **(C1,C2)** The influence of conditions and options on the percentage of preferences of the variable option **(C1)** and intermediate option **(C2)** in CE problems. **(D1,D2)** The influence of conditions and options on the percentage of preferences of the variable option **(D1)** and intermediate option **(D2)** in RE problems.

First, we investigated the effects of conditions and options on the percentage of allocation to the variable option in the CE problems. In the CE problems, there was a significant main-effect of conditions on the percentage of allocations to the variable option [description: 37% < experience: 50%; *F*(1,156) = 6.96, *p* = 0.01, $\eta_{p}^{2}$ = 0.04]. However, the main-effect of options on the percentage of allocations to the variable option was not significant [two-option: 46% ∼ three-option: 41%; *F*(1,156) = 0.95, *p* = 0.33, $\eta_{p}^{2}$ = 0.01]. Furthermore, the interaction effect of conditions and options on the percentage of allocations to the variable option was not significant [*F*(1,156) = 1.87, *p* = 0.17, $\eta_{p}^{2}$ = 0.01]. **Figure 7(A1)** shows the influence of conditions and options on the percentage of allocation to the variable in the CE problems.

Next, we investigated the effects of conditions and options on the percentage of allocation to the intermediate option in the CE problems. The main-effect of conditions on the percentage of allocation to the intermediate option was not significant [description: 14% ∼ experience: 17%; *F*(1,156) = 1.04, *p* = 0.31, $\eta_{p}^{2}$ = 0.01]. There was a significant main-effect of options on the percentage of allocations to the intermediate option [two-option: 0% < three-option: 31%; *F*(1,156) = 99.17, *p* < 0.001, $\eta_{p}^{2}$ = 0.39]. Furthermore, the interaction effect of conditions and options on the percentage of allocations to the intermediate option was not significant [*F*(1,156) = 1.04, *p* = 0.31, $\eta_{p}^{2}$ = 0.01]. **Figure 7(A2)** shows the influence of conditions and options on the percentage of allocation to the intermediate option in the CE problems.

Then, we investigated the effects of conditions and options on the percentage of allocation to the variable option in the RE problems. There was a significant main-effect of conditions on the percentage of allocations to the variable option [description: 35% > experience: 21%; *F*(1,156) = 9.56, *p* < 0.001, $\eta_{p}^{2}$ = 0.06]. In addition, there was a main-effect of options on the percentage of allocations to the variable option [two-option: 41% > three-option: 15%; *F*(1,156) = 30.21, *p* < 0.001, $\eta_{p}^{2}$ = 0.16]. Furthermore, there was a significant interaction effect of conditions and options on the percentage of preferences of the variable option [*F*(1,156) = 11.03, *p* < 0.001, $\eta_{p}^{2}$ = 0.07]. **Figure 7(B1)** shows the influence of conditions and options on the percentage of allocation to the variable option in the RE problems.

Next, we investigated the effects of conditions and options on the percentage of allocation to the intermediate option in the RE problems. The main-effect of conditions on the percentage of allocation to the intermediate option was not significant [description: 13% ∼ experience: 19%; *F*(1,156) = 2.75, *p* = 0.10, $\eta_{p}^{2}$ = 0.02]. There was a significant main-effect of options on the percentage of allocation to the intermediate option [two-option: 0% < three-option: 31%; *F*(1,156) = 69.29, *p* < 0.001, $\eta_{p}^{2}$ = 0.31]. Furthermore, the interaction effect of conditions and options on the percentage of allocation to the intermediate option was not significant [*F*(1,156) = 2.75, *p* = 0.10, $\eta_{p}^{2}$ = 0.02]. **Figure 7(B2)** shows the influence of conditions and options on the percentage of allocation to the intermediate option in the RE problems.

The DE gap on the variable option was negative and similar between the two- and three-option CE problems. Furthermore, the DE gap on the intermediate option was not present in both the two- and three-option CE problems. The DE gap on the variable option was positive for the two-option RE problem; however, it became slightly negative for the three-option RE problem. Furthermore, the DE gap on the intermediate option was not present in both the two- and three-option RE problems. Across both experience and description conditions, the percentage of allocations to the variable option were either constant (CE problem) or decreased (RE problem) from the two-option problems to the three-option problems. However, the percentage of allocations to the intermediate option increased in both CE and RE problems. Overall, these results show that the DE gap on the allocation percentages to variable option in two- and three-option CE problems and two-option RE problems was similar to that reported in literature (Hertwig et al., 2004). However, the DE gap was different from literature in the three-option RE problem.

Next, we evaluated the DE gap in terms of choice preferences for the variable option in the CE problems. There was a significant main-effect of conditions on the percentage of preferences to the variable option [description: 35% < experience: 59%; *F*(1,156) = 9.44, *p* < 0.001, $\eta_{p}^{2}$ = 0.06]. However, the main-effect of options on the percentage of preferences to the variable option was not significant [two-option: 50% ∼ three-option: 44%; *F*(1,156) = 0.66, *p* = 0.42, $\eta_{p}^{2}$ = 0.01]. Furthermore, the interaction effect of conditions and options on the percentage of preferences to the variable option was not significant [*F*(1,156) = 0.65, *p* = 0.42, $\eta_{p}^{2}$ = 0.01]. **Figure 7(C1)** shows the influence of conditions and options on the percentage of preferences of the variable option in the CE problems.

Then, we investigated the DE gap in terms of choice preferences for the intermediate option in the CE problems. The main-effect of conditions on the percentage of preferences of the intermediate option was not significant [description: 12% ∼ experience: 19%; *F*(1,156) = 2.15, *p* = 0.15, $\eta_{p}^{2}$ = 0.01]. There was a significant main-effect of options on the percentage of preferences of the intermediate option [two-option: 0% < three-option: 30%; *F*(1,156) = 34.35, *p* < 0.001, $\eta_{p}^{2}$ = 0.18]. Furthermore, the interaction effect of conditions and options on the percentage of preferences of the intermediate option was not significant [*F*(1,156) = 2.15, *p* = 0.15, $\eta_{p}^{2}$ = 0.01]. **Figure 7(C2)** shows the influence of conditions and options on the percentage of preferences of the intermediate option in the CE problems.

In the RE problems, there was a significant main-effect of conditions on the percentage of preferences of the variable option [description: 38% > experience: 19%; *F*(1,156) = 7.91, *p* = 0.01, $\eta_{p}^{2}$ = 0.05]. In addition, there was a main-effect of options on the percentage of preferences of the variable option [two-option: 38% > three-option: 19%; *F*(1,156) = 7.91, *p* = 0.01, $\eta_{p}^{2}$ = 0.05]. Furthermore, the interaction effect of conditions and options on the percentage of preferences of the variable option was significant [*F*(1,156) = 10.16, *p* < 0.001, $\eta_{p}^{2}$ = 0.06]. **Figure 7(D1)** shows the influence of conditions and options on the percentage of preferences of the variable option in the RE problems.

Next, we investigated the effects of conditions and options on the percentage of preferences of the intermediate option in the RE problems. The main-effect of conditions on the percentage of preferences of the intermediate option was not significant [description: 08% ∼ experience: 17%; *F*(1,156) = 3.44, *p* = 0.06, $\eta_{p}^{2}$ = 0.02]. There was a significant main-effect of options on the percentage of preferences of the intermediate option [two-option: 0% < three-option: 24%; *F*(1,156) = 25.37, *p* < 0.001, $\eta_{p}^{2}$ = 0.14]. Furthermore, the interaction effect of conditions and options on the percentage of preferences of the intermediate option was not significant [*F*(1,156) = 3.44, *p* = 0.06, $\eta_{p}^{2}$ = 0.02]. **Figure 7(D2)** shows the influence of conditions and options on the percentage of preferences of the intermediate option in the RE problems.

Overall, the DE gap on the variable option in the two- and three-option CE problems was negative. Furthermore, the DE gap on the intermediate option was not present in both the two- and three-option CE problems. In the RE problems, the DE gap on the variable option was positive for the two-option problem; however, it was slightly negative for the three-option problem. Furthermore, the DE gap on the intermediate option was not present in both the two- and three-option RE problems. Across both experience and description conditions, the percentage of preferences of the variable option were either constant (CE problem) or decreased (RE problem) from the two-option problems to the three-option problems. However, the percentage of preferences of the intermediate option increased from two-option problems to three-option problems in both CE and RE problems. Overall, these results show that the DE gap on the preference percentages to variable option in two- and three-option CE problems and two-option RE problems was similar to that reported in the literature (Hertwig et al., 2004). However, the DE gap was different from literature in the three-option RE problem.

#### Influence of Choice-Set Size on Switching Behavior

**FIGURE 8:**
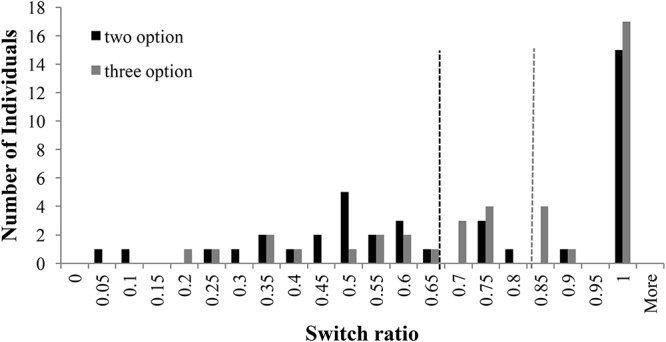
Switch ratio versus number of individuals across options in the CE problem. The dashed line in black represents the median switch ratio (=0.67) for the two-option CE problem; whereas, the dashed line in grey represents the median switch ratio (=0.84) for the three-option CE problem.

The choice-set size is likely to influence switching between options. Using the same methodology as in Experiment 1, we analyzed the distribution of switch ratio in two-option and three-option CE and RE problems. **Figure 8** shows the distribution of switch-ratios in two- and three-option CE problems. For the two-option CE problems, the plot showed two peaks at 0.50 and 1.0 switch ratios tending toward a bi-modal distribution. However, for the three-option CE problem, the peak at 0.50 shifted forward to two equally high peaks at 0.75 and 0.85 switch ratios, respectively. Furthermore, there was a forward shift in the median between two-option and three-option problems: the median at 0.67 in two-option CE problem shifted to 0.84 in three-option CE problem. Overall, the introduction of the intermediate option caused participants to switch more between options in the three-option CE problem compared to the two-option CE problem.

**FIGURE 9:**
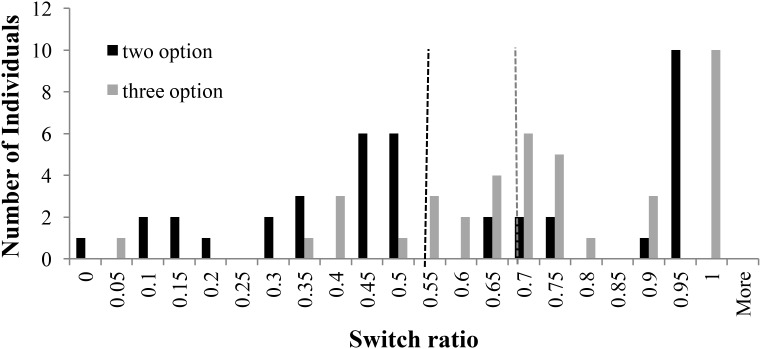
Switch ratio versus number of individuals across options in the RE problem. The dashed line in black represents the median switch ratio (=0.53) for the two-option RE problem; whereas, the dashed line in grey represents the median switch ratio (=0.67) for the three-option RE problem.

**Figure 9** shows the distribution of switch-ratios in two- and three-option RE problems. For the two-option RE problem, the histogram showed two equally high peaks at 0.45 and 0.50 switch ratios and another peak at 0.95 switch ratio. Thus, the two-option RE problem exhibited an approximate bi-modal distribution. However, in the three-option RE problem, the peaks at 0.45 and 0.50 shifted to 0.7 switch ratio and the peak at 0.95 shifted to 1.00 switch ratio. Furthermore, there was again a forward shift in the median between two-option and three-option problems: the median at 0.53 in two-option RE problem shifted to 0.67 in three-option RE problem. Thus, the introduction of the intermediate option caused people switch more between options in the three-option RE problem compared to the two-option RE problem.

#### Limited Information Search

**FIGURE 10:**
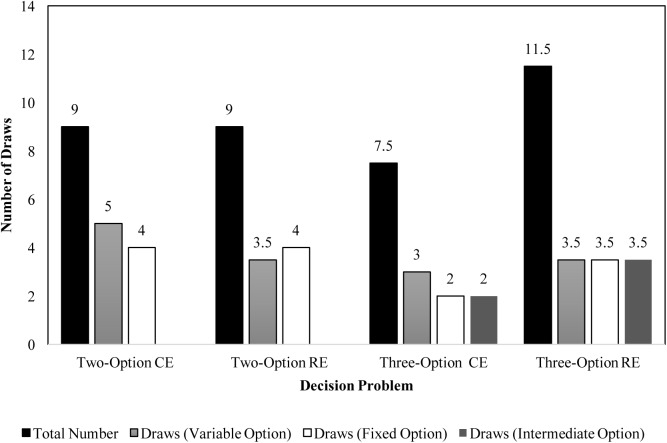
Median number of draws during sampling in two- and three-option common and rare problems in the experience condition.

As explained above, participants are likely to underweight rare outcomes due to small sample sizes. To investigate underweighting of rare outcomes due to small samples, we analyzed the median sample size across different problems. **Figure 10** displays the median number of draws of different options in two- and three-option CE and RE problems in the experience condition. In both the two-option problems, like Experiment 1, the median sample size value was similar to that reported in literature (Hertwig et al., 2004; Weber et al., 2004). Furthermore, as seen in **Figure 10**, the total number of median draws were higher for three-option RE problem compared to its two-option RE counterpart. Participants selected the intermediate option a sizable number of times in the three-option RE problem and the median sample sizes for the fixed and variable options were similar between two-option and three-option RE problems. In contrast, the total number of median draws were lesser in the three-option CE problem compared to its two-option counterpart. Also, the median number of samples of fixed and variable options were lesser in the two-option CE problem compared to the three-option CE problem. Again, participants selected the intermediate option a sizable number of times in the three-option CE problem. The reversal in sign of the DE gap in allocations and preferences between two-option and three-option RE problems could be attributed to the increase in the sample size between these problems.

#### Frequency Effects

To test the role of frequency in the current experiment, we analyzed how choices were influenced by the frequency of observing rare or high outcomes (**Table 3**). For the two-option CE problem, when the rare outcome (0.88) was encountered less frequently than expected, participants selected the variable option 81% of the time. However, when this rare outcome was encountered as or more frequently than expected, participants selected the variable option only 54% of the time. Interestingly, this pattern became stronger in the three-option CE problem: Participants selected the variable option 59% (38%) of the time when it was encountered less frequently (as or more frequently) than expected. For the intermediate option, as per our expectation, when the high outcome was encountered as or more frequently than expected about 57% of participants selected the intermediate option. In contrast, only 12% of participants selected the intermediate option when the high outcome in this option was encountered less frequently than expected. These observations resulted in the similarity of the DE gap between the two-option and three-option CE problems and the increase in percentage of preferences and allocations to the intermediate option. In the two-option RE problem, when the rare outcome (3.28) was encountered less frequently than expected, participants selected the variable option 11% of the time. However, when the rare outcome was encountered as expected or more frequently than expected, participants selected the variable option 31% of the time. This pattern was again intensified in the three-option RE problem: Participants selected the variable option 4% of the time when the rare outcome was encountered less frequently than expected; however, they selected the option with rare outcome 38% of the time when it was encountered as expected or more frequently than expected. For the intermediate option in the three-option RE problem, when the high outcome was encountered as or more frequently than expected about 50% of participants selected the intermediate option. In contrast, only 15% of participants selected the intermediate option when the high outcome in this option was encountered less frequently than expected. Overall, these results accounted for a smaller (greater) percentage of preferences and allocations to the variable (intermediate) option in the RE problems.

**Table 3:** Percentage of respondents in the experience condition who selected the option involving the rare and high outcomes in problems as a function of how often these rare and high outcomes were encountered during sampling.

Decision problem	Options			Rare/high outcome	Percentage choosing variable/intermediate option	
	Variable	Fixed	Intermediate		Encountered as frequently as or more frequently than expected	Encountered less frequently than expected
Two-option CE	1.18, 0.8; 0.88, 0.2	1.1, 1		0.88, 0.2 (Rare)	54 (13/24)	81 (13/16)
Three-option CE	1.18, 0.8; 0.88, 0.2	1.1, 1		0.88, 0.2 (Rare)	38 (05/13)	59 (16/ 27)
			1.34, 0.5; 0.88, 0.5	1.34, 0.5 (High)	57 (13/23)	12 (02/17)
Two-option RE	3.28, 0.1; 0.88, 0.9	1.1, 1		3.28, 0.1 (Rare)	31 (04/13)	11 (03/27)
Three-option RE	3.28, 0.1; 0.88, 0.9	1.1, 1		3.28, 0.1 (Rare)	38 (06/13)	04 (02/27)
			1.34, 0.5; 0.88, 0.5	1.34, 0.5 (High)	50 (10/20)	15 (03/20)

#### Recency Effects

As discussed in the experiment above, recency effects have been implicated in the DE gap, where recently sampled outcomes receive greater weight than outcomes not sampled recently (Hertwig and Pleskac, 2010; Dutt and Gonzalez, 2012; Lejarraga et al., 2012). As shown in **Table 4**, overall, the three-option RE and CE problems exhibited a greater recency effect compared to their two-option counterparts. For example, although there was no recency effect and a weak recency effect among the two-option CE and RE problems (CE: 63–63% = 0%; RE: 73–60% = 13%); however, the recency effect was much stronger among the three-option CE and RE problems (CE: 63–50% = 13%; RE: 70–45% = 25%). This reliance on recency in three-option problems likely caused people to choose more often the intermediate option.

**Table 4:** Recency effects in two- and three-option common and rare problems in the experience condition.

Decision problem	Percentage of actual choices that coincided with predicted choice	
	First half	Second half
Two-option CE	63 (25/40)	63 (25/40)
Three-option CE	60 (24/40)	73 (22/40)
Two-option RE	50 (20/40)	63 (25/40)
Three-option RE	45 (18/40)	70 (28/40)

### Natural Mean Heuristic (NMH) Model

**FIGURE 11:**
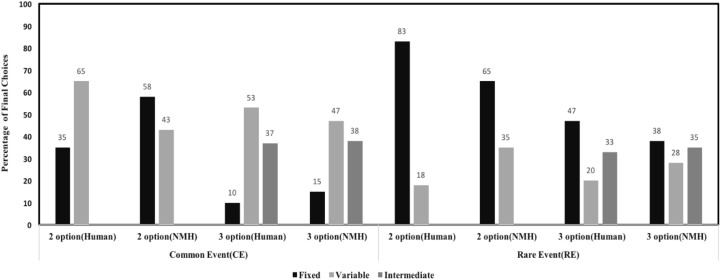
Percentage of final choices for each option by human participants and NMH model.

As explained above, the NMH model seeks to capture sample size, outcome frequency, and outcome magnitude for predicting choices. **Figure 11** shows model comparison between NMH model and human data. For two-option CE problems, the MSD between human data and model across the different options was 0.098. For the three-option CE problem, MSDs between human data and model data across the different options was 0.0061. For the two-option RE problem, the MSD between human data and model across different options was 0.0724. In three-option RE problem MSD between human data and model across the different options was 0.0254. Barring the two-option CE problem, the NMH model could account for human choices across all other problems.

### Discussion

In this experiment, we investigated the influence of options (two or three) and conditions (experience or description) on the percentage of allocations and preferences across both rare and common event problems without the investment framing. Our results showed that the DE gap on the variable option in two- and three-option CE problems and two-option RE problems was similar to that reported in the literature (Hertwig et al., 2004). However, in agreement with Noguchi and Hills (2016), the DE gap on the variable option was different from literature in the three-option RE problem. Also, the preference and allocation percentages to the variable option were either constant (CE problem) or decreased (RE problem) from the two-option problems to the three-option problems. However, the preference and allocation percentages to the intermediate option increased in both CE and RE problems.

First, we found that the DE gap persisted in two- and three-option CE problems and two-option RE problems. A likely reason for this finding is the underweighting of rare events in these problems, which was evident from the frequency analyses (Hertwig and Pleskac, 2010; Dutt and Gonzalez, 2012; Lejarraga et al., 2012). For the two-option CE problem, when the rare outcome (0.88) was encountered less frequently than expected, a large majority of participants selected the variable option. However, when this rare outcome was encountered as or more frequently than expected, fewer percentage of participants selected the variable option. This pattern became stronger in the three-option CE problem. Similarly, in both the two- and three-option RE problems, when the rare outcome (3.28) was encountered less frequently than expected, a smaller percentage of participants selected the variable option compared to when the rare outcome was encountered as expected or more frequently than expected. These patterns reveal that participants underweighted the rare events across all problems.

However, we also found a slight reversal of the DE gap in the three-option RE problem. As participants seem to underweight the rare outcome (3.28) in this problem in experience, the reversal of the gap is likely due to the lack of over-weighting of the rare outcome in the description condition. It is likely that in the description condition, participants were attracted to the intermediate and fixed options in this problem. The attraction to the intermediate option could be due to the fact that this option provided the next highest outcome with a 50% chance. In addition, the attractiveness to the fixed option could be because this option provided the third highest outcome (1.1) with a 100% chance.

We also found that a significant increase in the allocations and preferences for the intermediate option from two-option problems to three-option problems and mostly this increase was on account of a decrease in the allocations and preferences for the variable option. For the intermediate option, when the high outcome (1.34) was encountered as or more frequently than expected a greater majority selected the intermediate option compared to when the high outcome in this option was encountered less frequently than expected. Also, the excessive reliance on recency as well as a movement to piecewise strategy in the three-option problems likely increased participants’ choices and allocations for the intermediate option. That is because in the presence of recency and switching, this option would be the most attractive in the RE problems (1.34 occurring more frequently than 3.28) and equally attractive in the CE problems (1.34 being greater in magnitude compared to 1.18).

Furthermore, we found that the NMH model (Hertwig and Pleskac, 2010) could account for human choices across all conditions except for the two-option common event problem. We observed that in the two-option common event problem, the number of samples of different options were much larger compared to those in the three-option common event problem. It is likely that the larger number of samples of different options adversely impacted the natural means in the NMH model and caused the model to not predict the choice percentage of the variable option more than those for the fixed option. Unlike the NMH model, the human decisions are also influenced by the recency of experienced outcomes. Thus, human choices do show the DE gap in the two-option common event problem even after excessive sampling of different options in this problem.

## General Discussion

Experiment 2 was a replication of Experiment 1, where we removed the investment framing from problems. Barring differences in behavior in one of the problems, overall, our results on the DE gap were similar between the two experiments and showed the robustness of the DE gap both in the presence and absence of the intermediate option.

However, there were certain important differences in results between the two experiments, which could be due to the difference in framing of problems across the two studies. First, there was a much stronger frequency effects created by the intermediate option’s high outcome in Experiment 1 compared to that created by the intermediate option’s high outcome in Experiment 2. Second, there were stronger recency effects created due to the presence of the intermediate option in Experiment 1 compared to the effects created due to this option’s presence in Experiment 2. Third, the number of sample of different options were larger in Experiment 2 compared to those in Experiment 1. Finally, the RE problem results in Experiment 2 were similar to those reported in Noguchi and Hills (2016); however, the RE problem results in Experiment 1 were different from those reported in Noguchi and Hills (2016). Overall, these observations, likely due to the presence of investment framing in Experiment 1 and its absence in Experiment 2, caused the reappearance of the DE gap on the intermediate option in the first experiment and its absence in the second experiment.

We also found that the NMH model could account for observations in human data, especially in three-option problems across both experiments. The NMH model incorporates the sample size, the outcome frequency, and the outcome magnitude in its working. The NMH model was able to explain human preferences across both experiments as it contained mechanisms that are likely implicated in influencing the decision-making process in experiment’s problems.

First, if a problem consists of making a binary choice, where one option is variable with a non-zero rare outcome and the other option is fixed with a constant outcome (the RE problem), then NMH suggests a choice for the constant option due to presence of a small sample size, frequency effects, and the different magnitudes of experienced outcomes. Now, if this problem consists of an additional intermediate option, where the probability of non-zero outcome is high and its magnitude is higher than the fixed option, then, according to the NMH model, people would underweight the non-zero rare outcome and discount the variable option (because of the sample size and frequency effects). Next, based upon the NMH procedure, while choosing between the fixed option and intermediate option, people would tend to overweight the high probability of the non-zero outcome in the intermediate option and choose the intermediate option as that would maximize their outcomes.

In contrast, if a problem consists of making a binary choice, where one option is variable with a non-zero frequent outcome and the other option is fixed with a constant outcome (the CE problem), then, according to the NMH model, the variable option is likely to be chosen (due to small a sample size and frequency effects that underweights the zero-valued rare outcome in the variable option). If this problem consists of an additional intermediate option, where the probability of non-zero outcome is high, then, according to NMH, people would tend to underweight the zero-valued rare outcome; and, discount the variable option compared to the intermediate option. This behavior will likely result if the non-zero outcome in the intermediate option is higher in magnitude compared to the non-zero outcome in the variable option. Next, according to NMH, while choosing between the fixed option and intermediate option, people would tend to overweight the high probability of non-zero outcome in the intermediate option and choose the intermediate option as their final choice. Again, this explanation dictates that a large percentage of participants, while making decisions from experience, are likely to choose the intermediate option compared to the variable and fixed options when options increase from two to three.

First, a likely reason for higher sampling in Experiment 2 compared to Experiment 1 could be because of the availability bias (Tversky and Kahneman, 1973) created by the “investment framing” compared to the “allocation framing.” According to the availability bias, people tend to heavily weigh their judgments toward more recent information, making new opinions biased toward information that is readily available to them (Tversky and Kahneman, 1973). In the real world, it is likely that people hear more about making investments from their family and friends compared to making allocations. Thus, the investment framing in experiment 1, being more available, caused people to form a quicker assessment about options compared to the allocation framing.

Second, Noguchi and Hills (2016) used several problems in their study, where the number of options varied between 2 and 32. This substantial increase in the number of options may have created significant variations in presentation and processing of visual information, task complexity, number of samples drawn (in the case of experience-based decisions), and working memory load. However, in our paper, we only increased the number of options in different problems by one (from two options to three options). Thus, our design seems to be more balanced regarding different mental, physical, and cognitive factors compared to the design of Noguchi and Hills (2016). However, it would be advisable to measure the influence of these factors across different option problems as an extension of this study. For example, one could use the NASA Task Loading Index (Hart and Staveland, 1988) to evaluate the additional mental, physical, and cognitive load due to the increase in the number of options.

Third, although there is a large body of literature documenting the existence of the DE gap (Gottlieb et al., 2007; Erev et al., 2010; Abdellaoui et al., 2011; Gonzalez and Dutt, 2011), one could question the equivalence of the description and experience conditions in these experiments. In the description condition, information about probability and outcomes is simultaneously presented to participants; whereas, in the experience condition, participants need to keep a running account of different outcomes and their frequency of occurrence (Hertwig et al., 2004). Thus, the experience condition, although being mathematically equivalent to the description condition, may put more load on participants’ working memory. Again, the differences between description and experience conditions regarding the pressure put on working memory need to be investigated as an extension of our study.

Also, in the second experiment, we used problem without the investment framing by changing the word “investment” to “allocation.” We would term Experiment 2 as a replication of our results in the first experiment, where conditions, problem types (CE or RE), and the number of options (two or three) remained constant across both studies. Thus, a natural extension of this study could be to try problems that are different from each other in the presence or absence of framing.

We plan to extend the current investigation in several ways as part of our future research. First, it might be worthwhile to increase the number of intermediate options further from one option to two or more to check whether the recency and frequency processes continue to influence choices and allocations across different problems and contextual framing. Next, it would be interesting to see how the intermediate option influences decisions when the problems are dynamic: They possess changing probabilities compared to stationary probabilities in the variable and intermediate options. Also, it would be interesting to evaluate whether the recency and frequency processes continue to influence choices when participants are forced to sample a fixed number of times. Still, it would be worthwhile to investigate how other computational models like Cumulative Prospect Theory (Tversky and Kahneman, 1992) and Instance-based Learning (Lejarraga et al., 2012) would account for the observed phenomena compared to the NMH model. These investigations form the immediate next steps for us to execute in our ongoing research program on decisions from experience.

## Author Contributions

NS: main contributor, data collection, and analysis. SD: prepared the initial draft. VD: overall supervision in carrying out research.

## Conflict of Interest Statement

The authors declare that the research was conducted in the absence of any commercial or financial relationships that could be construed as a potential conflict of interest.

## Footnotes

1 An option is asymmetrically dominated when it is inferior to some options and it is superior to other options.

2 Literature has also proposed a repeated choice framework within decisions from experience, where each decision choice is consequential (Abdellaoui et al., 2011).
